# Sources of bias in measures of allele-specific expression derived from RNA-seq data aligned to a single reference genome

**DOI:** 10.1186/1471-2164-14-536

**Published:** 2013-08-07

**Authors:** Kraig R Stevenson, Joseph D Coolon, Patricia J Wittkopp

**Affiliations:** 1 Department of Computational Medicine and Bioinformatics, University of Michigan, Ann Arbor, MI 48109, USA; 2 Department of Ecology and Evolutionary Biology, University of Michigan, Ann Arbor, MI 48109, USA; 3 Department of Molecular, Cellular, and Developmental Biology, University of Michigan, 830 North University Avenue, Ann Arbor, MI 48109, USA

**Keywords:** Next-generation sequencing, Mapping bias, *Drosophila melanogaster*, *Drosophila simulans*, DGRP, Allelic imbalance, Genomics, Gene expression, Illumina

## Abstract

**Background:**

RNA-seq can be used to measure allele-specific expression (ASE) by assigning sequence reads to individual alleles; however, relative ASE is systematically biased when sequence reads are aligned to a single reference genome. Aligning sequence reads to both parental genomes can eliminate this bias, but this approach is not always practical, especially for non-model organisms. To improve accuracy of ASE measured using a single reference genome, we identified properties of differentiating sites responsible for biased measures of relative ASE.

**Results:**

We found that clusters of differentiating sites prevented sequence reads from an alternate allele from aligning to the reference genome, causing a bias in relative ASE favoring the reference allele. This bias increased with greater sequence divergence between alleles. Increasing the number of mismatches allowed when aligning sequence reads to the reference genome and restricting analysis to genomic regions with fewer differentiating sites than the number of mismatches allowed almost completely eliminated this systematic bias. Accuracy of allelic abundance was increased further by excluding differentiating sites within sequence reads that could not be aligned uniquely within the genome (imperfect mappability) and reads that overlapped one or more insertions or deletions (indels) between alleles.

**Conclusions:**

After aligning sequence reads to a single reference genome, excluding differentiating sites with at least as many neighboring differentiating sites as the number of mismatches allowed, imperfect mappability, and/or an indel(s) nearby resulted in measures of allelic abundance comparable to those derived from aligning sequence reads to both parental genomes.

## Background

During the last five years, massively parallel sequencing of cDNA libraries synthesized from RNA samples (known as “RNA-seq”) has largely replaced the use of microarrays for comparative studies of gene expression (e.g. [1-3]). Advantages of RNA-seq over microarrays include a greater dynamic range and the ability to survey expression in new strains and species without the set-up costs of microarrays and without complications from hybridization differences among genotypes [4,5]. In addition, because RNA-seq provides full sequence information for the transcriptome, it is better suited for discovering novel transcripts and splice isoforms and for quantifying allelic abundance in heterozygous and mixed genotype samples than microarrays. Measures of allele-specific expression (ASE) are particularly important for studying the regulation of gene expression because they can be used to distinguish *cis*- and *trans*-regulatory changes [6,7] and to detect genomic imprinting [8,9].

To quantify transcript abundance using RNA-seq, each short sequence read (hereafter simply called a “read”) is compared to an annotated reference genome. Assignment of a read to a specific gene is made by finding the region of the genome with the highest sequence similarity, and the number of reads aligning to a gene is used as a proxy for its relative expression level [4]. Mapping reads to specific genes is relatively straightforward with the bioinformatics tools available today [10-13], but using these tools to distinguish between reads derived from alternative alleles of the same gene remains challenging [9]. This challenge was most clearly demonstrated by Degner et al. [14], who simulated reads from a heterozygous human genotype and assigned them to specific alleles after mapping to a reference human genome. Reads perfectly matching the reference genome were assigned to the reference allele, whereas reads containing mismatches to the reference genome were assigned to the alternative allele. Despite simulating an equal number of reads from each allele, a bias was observed causing reads to be assigned more often to the reference allele than the alternative allele. Controlling for sites known to be polymorphic in humans prior to aligning the simulated reads produced symmetrical measures of relative ASE, showing that the differentiating sites themselves caused this bias.

Recently, two alternative strategies for aligning reads have been shown to eliminate the systematic bias in measures of relative ASE favoring the reference allele. In the first, RNA-seq reads are aligned separately to maternal and paternal genomes. These allele-specific genomes can be generated either by sequencing inbred lines with the maternal and paternal genotypes [8,15-17] or by inferring the maternal and paternal haplotypes using phased genotype information such as that available for humans from the 1000 Genomes Project Consortium [18,19]. However, researchers interested in measuring relative ASE in organisms for which parent-specific genomes cannot be readily obtained will struggle to use this approach. The second strategy is to consider all possible phasings of variants that can occur in the same sequence read and either supplement the reference genome with these haplotypes [20] or use this information during alignment with a polymorphism-aware aligner, such as GSNAP [21,22]. This is a viable strategy for both model and non-model species, but will likely be most effective for intraspecific studies of species like humans with relatively low levels of polymorphism because the number of possible haplotypes increases exponentially with the number of polymorphic sites.

To better understand the source(s) of biased measures of relative ASE, we identified properties of sites showing inaccurate measures of relative ASE using simulated *Drosophila* sequencing data with known values of relative allelic abundance. Simulated datasets contained either ~10-fold or ~100-fold more differentiating sites than the human genotypes used to validate other methods for measuring relative ASE [14,18,20]. We also examined the impact of these factors on measures of relative ASE derived from real sequencing data. Reads from simulated and real sequencing data were aligned to a single reference genome, varying the number of mismatches allowed, as well as aligned to separate maternal and paternal genomes with no mismatches allowed. We found that limiting analysis of relative ASE to regions of the genome with no more differentiating sites than the number of mismatches allowed eliminated the systematic bias toward the reference allele and produced measures of ASE similar to those inferred from aligning reads separately to the maternal and paternal genomes. Excluding differentiating sites contained within reads that cannot be aligned uniquely or that overlap an insertion or deletion (indel) further improved measures of relative allelic abundance.

## Results and discussion

### The systematic bias in measures of ASE correlates with the density of differentiating sites

As described above, Degner et al. [14] found that allele-specific reads mapped preferentially to the reference allele when using a single reference genome to quantify ASE. The alignment parameters they used allowed two or fewer bases within each read to differ from the reference genome. Reads perfectly matching the reference genome were assigned to the reference allele, while reads with at least one difference from the reference genome were assigned to an alternative allele. We hypothesized that the inability to map reads with more differences from the reference genome than mismatches allowed underestimated the abundance of the alternative allele and caused measures of ASE to be biased toward the reference allele.

**Figure 1:**
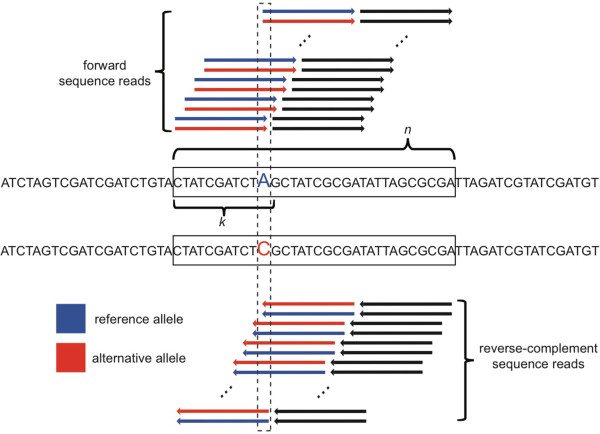
**Simulating an allele-specific RNA-seq experiment.** Reads were generated from the “reference” *D. melanogaster* (*dm3*) allele (blue) and from an “alternative” allele (red) that contained all homozygous single nucleotide variants found in the DGRP strain “line_40”. For each exon, one read (arrow) was generated starting at each position for each allele from 1 to *n-k*, where *n* is the length of the exon and *k* is the length of the read, both in bases. This process was repeated for the reverse complement of each exon. The black arrows indicate reads with no allele-specific information.

To test this hypothesis, we generated an equal number of reads from two genotypes *in silico*, combined them, and measured the relative abundance of allele-specific reads. These sequences were derived from 52,370 non-overlapping constitutively-expressed exons in *Drosophila melanogaster* (Additional file 1; [15]). The annotated *D. melanogaster* genome (*dm3*) was used as the “reference” allele, and an edited version of this genome with 93,781 coding sites altered to match alleles in a line of *D. melanogaster* from the Drosophila Genetic Reference Panel [23,24] was used as an “alternative” allele. We generated 36-base reads from each allele starting at every possible position in each exon and repeated this process for both strands of DNA because RNA-seq is usually performed using double-stranded cDNA (Figure 1). This process generated 93,395,272 reads, representing ~3.4 Gb of sequencing data. Importantly, this approach guaranteed that reads from each allele were present in equal amounts. To quantify relative allelic abundance as a proxy for relative ASE, we aligned each read to the reference genome using Bowtie [10], excluding reads that mapped to multiple locations, and evaluated the number of reads assigned to the reference and alternative alleles at each differentiating site using SAMtools [13].

**Figure 2:**
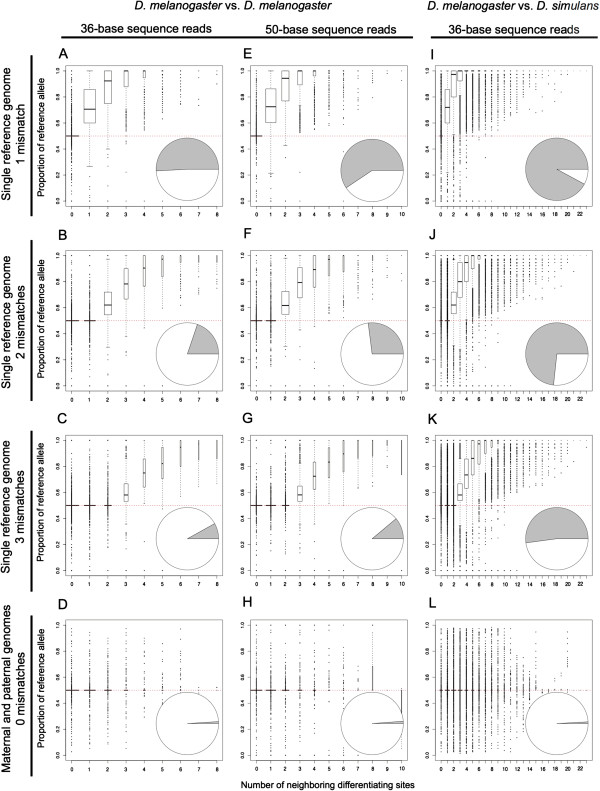
**The density of differentiating sites affects relative allelic abundance when simulated reads are mapped to only one genome.** Relative allelic abundance was measured using the 36-base **(A-D)** and 50-base **(E-H)** reads simulated from the two *D. melanogaster* genotypes as well as using the 36-base reads simulated from *D. melanogaster* and *D. simulans***(I-L)** aligned to a single reference genome, allowing either one mismatch **(A, E, I)**, two mismatches **(B, F, J)**, or three mismatches **(C, G, K)**, as well as by aligning reads to both allele-specific genomes allowing no mismatches **(D, H, L)**. The number of neighboring differentiating sites is shown on the x-axis of each panel for each differentiating site and describes the maximum number of other sites that differ between the two alleles in any potential read overlapping the focal differentiating site. The y-axis shows the proportion of reads that were assigned to the reference allele for each differentiating site, summarized in box plots where the width of each box is proportional to the number of sites in that class. A proportion of 0.5 (indicated with a red dotted line in each panel) is expected if all reads overlapping a differentiating site are correctly assigned to alleles. The pie chart inset in each panel shows the total number of differentiating sites with equal (white) and unequal (grey) abundance of reads assigned to each allele.

Initially, we allowed one mismatch to the reference genome during the alignment step, which is the minimum number required to align a read from the alternative allele. We found that 50.9% of differentiating sites had unequal measures of allelic abundance, 99.3% of which were biased toward the reference allele. To determine whether this bias was influenced by the density of differentiating sites, we calculated the maximum number of sites that differed between the two alleles among all possible 36-base reads overlapping each differentiating site (Figure 1). Of all sites considered, 49.8% had at least one neighboring differentiating site (i.e., at least one other differentiating site within an overlapping read). Of these sites, 99.8% showed more reads assigned to the reference allele than to the alternative allele. Furthermore, the extent of bias toward the reference allele increased with the number of neighboring differentiating sites (Figure 2A). This bias was caused by the failure of reads simulated from the alternative allele to align to the reference genome more often than those simulated from the reference allele. Aligning reads to only the alternative allele produced complementary results (Additional file 2). These findings are consistent with our hypothesis that the density of differentiating sites complicates the mapping of reads and leads to biased measures of relative ASE.

To decrease the impact of neighboring differentiating sites on allelic assignment, we allowed two or three mismatches when aligning our simulated reads to the reference genome. We found that increasing the number of mismatches improved measures of allelic abundance: 80.2% and 91.9% of differentiating sites were inferred to be equally abundant when two and three mismatches, respectively, were allowed. A bias toward the reference allele was still observed, but only for sites where the number of neighboring differentiating sites was greater than or equal to the number of mismatches allowed during the alignment step (Figure 2B,C). Increasing the number of mismatches allowed reduced the bias toward the reference allele, but increased the percentage of reads that failed to map uniquely: allowing one, two, and three mismatches, 2.2%, 2.5%, and 2.9% of all reads failed to map uniquely, respectively.

For comparison, we aligned the simulated reads independently to the reference and alternative genomes with the same parameters used when aligning reads to the single reference genome except that zero mismatches were allowed. This is analogous to aligning reads to the maternal and paternal genomes, which is a strategy that has previously been shown to produce unbiased measures of relative ASE [15,18-20,25]. We found that 99.0% of differentiating sites showed equal representation of the two alleles, with the rest showing no systematic bias toward either allele (Figure 2D). Only 1.9% of all reads were excluded because they failed to map uniquely to at least one genome.

### Read length and the amount of sequence divergence can also affect allelic bias

Given the observed impact of neighboring differentiating sites on allelic assignments, we hypothesized that longer reads might produce less accurate measurements of allele-specific abundance because they should overlap more neighboring differentiating sites. To test this hypothesis, we repeated our simulation with 50-base reads, determining the maximum number of sites that differed between the two alleles among all possible 50-base reads overlapping each differentiating site. We found that 40.6%, 73.0%, and 88.9% of differentiating sites showed equal representation of the two alleles when aligned to a single reference genome with one, two or three mismatches allowed (Figure 2E-G). Increasing the number of mismatches allowed when aligning the 50-base sequence reads to be more similar to the ratio of mismatches allowed for the 36-base sequence reads eliminated this difference, however. 91.9% and 92.1% of differentiating sites showed equal allelic abundance for 36- and 50-base reads when three and four mismatches, respectively, were allowed (Additional file 3). By contrast, 98.8% of differentiating sites showed equal representation when reads were aligned to the maternal and paternal genomes with zero mismatches allowed (Figure 2H).

Increased sequence divergence is also expected to affect measures of relative allelic abundance because it should increase the average number of neighboring differentiating sites within each read. To test this hypothesis, we simulated 36-base reads from two different *Drosophila* species *(D. melanogaster* and *D. simulans;*[16]) and analyzed them as described above, using the *D. melanogaster* exome as the single reference genome. Sequences from 60,040 orthologous exons with 1,130,435 differentiating sites were used for this simulation, which is an order of magnitude more differentiating sites than between the two strains of *D. melanogaster* analyzed. As predicted, we found that the bias toward the reference allele was higher for the interspecific comparison than for the intraspecific comparison when reads were aligned to a single reference genome (Figure 2, compare I-K with A-C). When aligning reads to both parental genomes, however, sequence divergence had a negligible impact: the intra- and interspecific datasets produced nearly identical results (Figure 2, compare L with D).

### Allele-specific differences in mappability and insertions/deletions affect measurements of ASE

Differences between alleles in sequences that appear more than once in the genome can also cause reads to be excluded for one allele but not the other [14]. Assuming the number of such differentiating sites is similar between alleles, differences in allele-specific mappability should not systematically favor one allele or the other, but will still cause errors in relative ASE. To examine the impact of mappability on measures of relative allelic abundance derived from our simulated data, we used software from the GEM library [26] to calculate a mappability score for each differentiating site by averaging the mappability scores of all possible reads that included that site. In each case, mappability scores were calculated using the same number of mismatches allowed during read alignment. Differentiating sites with an average mappability score < 1 were considered to have imperfect mappability when using a single reference genome. When using parental genomes, we summed the average mappability scores for each allele, and mappability scores < 2 were considered to have imperfect mappability.

**Figure 3:**
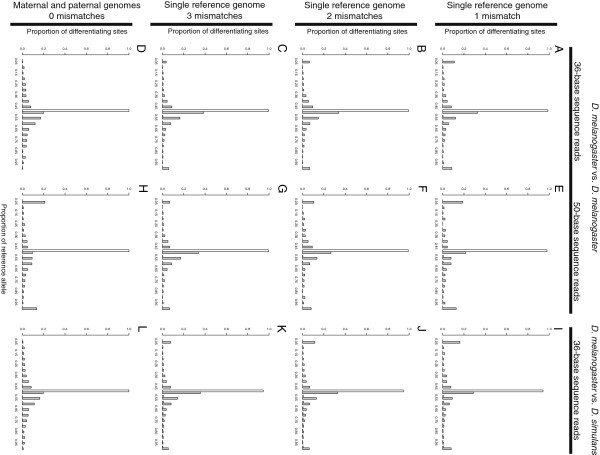
**Imperfect mappability causes inaccurate measures of relative allelic abundance.** For unbiased differentiating sites (i.e., those with fewer neighboring differentiating sites than the number of mismatches allowed) with either perfect (white) or imperfect (grey) mappability, the distribution of relative allelic abundance (measured as the proportion of mapped reads assigned to the reference allele) is shown for the 36-base **(A-D)** and 50-base **(E-H)** reads simulated from the two *D. melanogaster* genotypes as well as for the 36-base reads simulated from *D. melanogaster* and *D. simulans***(I-L)** aligned to a single genome, allowing one **(A, E, I)**, two **(B, F, J)**, or three **(C, G, K)** mismatches. The distribution of relative allelic abundance for unbiased differentiating sites with perfect (white) and imperfect (grey) mappability is also shown for all three simulated datasets after aligning reads to both the reference and alternative genomes, allowing no mismatches **(D, H, L)**.

We then compared relative allelic abundance for sites with perfect and imperfect mappability in all three simulated datasets (Figure 3), excluding sites with more neighboring differentiating sites than the number of mismatches allowed when aligning to a single reference genome. For both the 36- and 50-base reads simulated from the two *D. melanogaster* genotypes, > 97.9% of sites with perfect mappability showed the expected equal abundance of the reference and alternative alleles under all mapping conditions (Figure 3A-H). For the 36-base reads simulated from the *D. melanogaster* and *D. simulans* genomes, 99.9% of sites with perfect mappability showed equal abundance when reads were aligned to both parental genomes (Figure 3L), but only ~94% of sites with perfect mappability showed such equal abundance when reads were aligned to a single (*D. melanogaster*) reference genome (Figure 3I-K).

**Figure 4:**
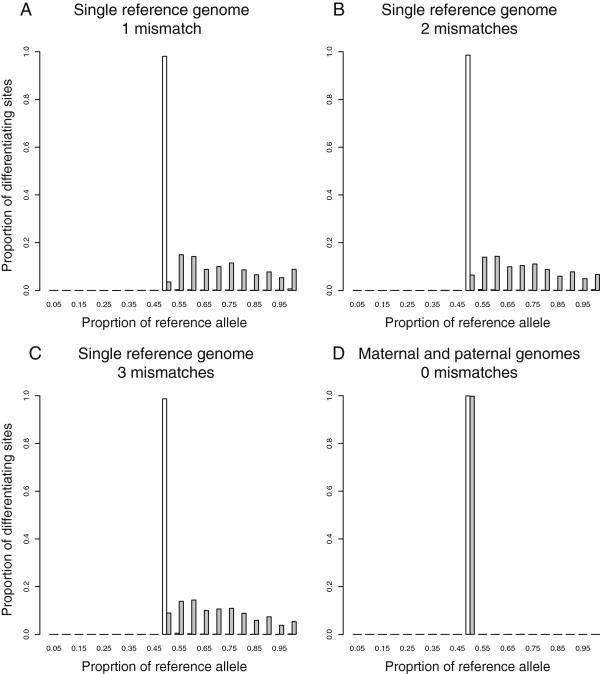
**Insertions and deletions (indels) cause biased allele-specific assignment when reads are aligned to a single reference genome.** For differentiating sites with perfect mappability and fewer neighboring differentiating sites than the number of mismatches allowed, the distributions of relative allelic abundance are shown for differentiating sites with (grey) and without (white) one or more nearby indel(s) after aligning the 36-base reads simulated from *D. melanogaster* and *D. simulans* to either the *D. melanogaster* genome with one **(A)**, two **(B)**, or three **(C)** mismatches allowed or to both the *D. melanogaster* and *D. simulans* genomes with no mismatches allowed **(D)**.

We hypothesized that this decrease in accuracy after aligning *D. melanogaster* and *D. simulans* reads to a single reference genome might be caused by the presence of insertions or deletions (indels) between *D. melanogaster* and *D. simulans* that are located near differentiating sites (i.e., within the length of a read from the differentiating site). Such indels can prevent the alignment of *D. simulans* reads to the *D. melanogaster* genome. Consistent with this hypothesis, we found that sites with perfect mappability that had an indel nearby showed more reads assigned to *D. melanogaster* than *D. simulans* allele when reads were aligned to only the *D. melanogaster* genome, whereas sites with perfect mappability that lacked such an indel did not (Figure 4A-C). When reads were aligned to both parental genomes, sites with perfect mappability showed equal representation of the two alleles regardless of the presence or absence of nearby indels (Figure 4D). Indels were not a factor in our comparisons of the two *D. melanogaster* strains because the alternative allele was constructed by changing only single nucleotides in the reference allele.

### Aligning real sequencing data to a single genome can produce reliable measures of relative ASE

Assessing the accuracy of relative ASE measurements derived from RNA-seq data is challenging because the true value of relative ASE is rarely known. Independent empirical methods for measuring relative ASE such as Pyrosequencing and qPCR can be used to validate RNA-seq data for individual genes, but they are not suitable for quantifying relative ASE on a genomic scale. Therefore, instead of using real RNA-seq data to evaluate factors affecting measures of relative ASE, we used sequence data that was collected in a comparable manner from genomic DNA extracted from F_1_ hybrids, in which all maternal and paternal alleles are expected to be present in equal amounts.

Specifically, we used 36-base reads from genomic DNA extracted from female F_1_ hybrids that were produced by crossing inbred strains of *D. melanogaster* and *D. simulans*[16]. These strains had the same genotypes as the *D. melanogaster* and *D. simulans* sequences used for the interspecific simulation described above. Reads were aligned to the *D. melanogaster* exons allowing one, two, or three mismatches, as well as to both the *D. melanogaster* and *D. simulans* exons allowing zero mismatches. Because real sequencing data involves stochastic sampling, the proportion of the reference allele observed was not always expected to be 0.5. Therefore, after aligning reads, we excluded differentiating sites with fewer than 20 overlapping reads and used binomial exact tests with a false discovery rate threshold of 0.05 to test each differentiating site for a statistically significant difference in relative allelic abundance [15,27].

**Figure 5:**
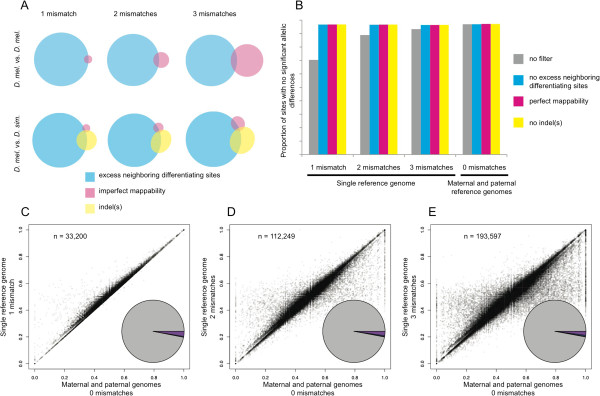
**Real reads aligned to a single reference genome produce reliable measures of allelic abundance after excluding problematic differentiating sites. (A)** The relative proportions of sites with an excess of neighboring differentiating sites (cyan), imperfect mappability (magenta), an indel(s) nearby (yellow), or more than one of these properties are shown for the simulated 36-base intra- (mel-mel) and interspecific (mel-sim) datasets allowing one (1 mm), two (2 mm), or 3 (3 mm) mismatches during alignment to a single reference genome. **(B)** The proportion of differentiating sites with no statistically significant difference in relative allelic expression is shown for the real reads from F1 hybrids between D. melanogaster and D. simulans after aligning to either a single reference genome with one, two, or three mismatches allowed or to both the maternal and paternal genomes with zero mismatches allowed before excluding any sites (grey) and after sequentially excluding differentiating sites with an excess of neighboring differentiating sties (cyan), imperfect mappability (magenta), or an indel(s) nearby (yellow). **(C-E)** For each differentiating site retained after filtering based on neighboring differentiating sites, mappability, and indels, the proportion of reads assigned to the reference allele is plotted after aligning reads to a single reference genome (y-axis) or to separate allele-specific genomes (x-axis), allowing one **(C)**, two **(D)**, or three **(E)** mismatches. The pie chart insets reflect the total number of differentiating sites that showed either no statistically significant difference in relative allelic abundance using either alignment strategy (grey), a statistically significant difference when reads were aligned to either a single reference genome (blue) or both the maternal and paternal genomes (red), or a significant difference with both alignment methods (purple). Binomial exact tests and a false discovery rate of 0.05 were used to assess statistical significance in all cases.

As described above, our simulated datasets showed that reads containing (1) as many or more neighboring differentiating sites as mismatches allowed during alignment, (2) imperfect mappability, and/or (3) an indel(s) between alleles can cause inaccurate measures of relative allelic abundance. Differentiating sites with an excess of neighboring differentiating sites were the most common of these three types of problematic sites in both intra- and interspecific simulations (Figure 5A). To determine the relative impact of each of these factors on measures of allele-specific abundance derived from real sequencing data, we filtered the differentiating sites based on each factor sequentially and determined the percentage of differentiating sites retained that had no statistically significant difference in abundance between alleles (hereafter referred to as “equal allelic abundance”) for each alignment strategy.

Prior to excluding any sites, 70.4%, 88.9%, and 93.3%, respectively, of all differentiating sites showed equal allelic abundance when reads were aligned to a single genome with one, two, or three mismatches allowed. After aligning reads to both parental genomes, 96.9% showed evidence of equal allelic abundance. Excluding differentiating sites with at least as many neighboring differentiating sites as the number of mismatches allowed increased this percentage to 96.3%-96.6% when aligning to a single reference genome (Figure 5B). Further restricting the set of differentiating sites to those with perfect mappability increased these percentages ~0.1%, and subsequently excluding differentiating sites with indels nearby increased the percentage of genes with equal allelic abundance an additional ~0.1% (Figure 5B). After filtering out these problematic sites, measures of relative allelic abundance derived from aligning reads to a single reference genome were similar to those produced by aligning sequence reads separately to the maternal and paternal genomes (Figure 5C-E).

### Excluding selected differentiating sites maintains ability to measure relative ASE for most exons

**Figure 6:**
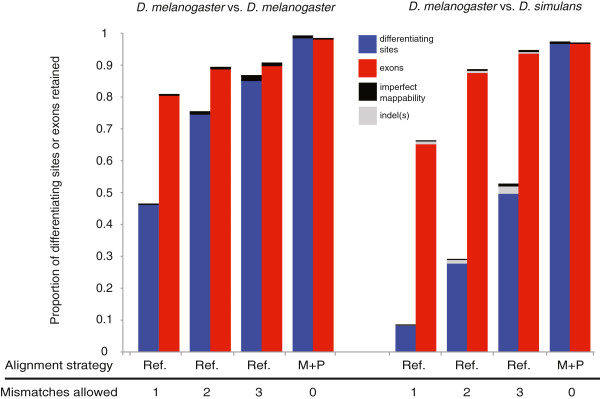
**Relative allelic abundance can be estimated for most exons after excluding sites problematic sites.** The proportion of differentiating sites (blue) and exons with at least one differentiating site (red) suitable for quantifying ASE after excluding sites with an excess of neighboring differentiating sites, imperfect mappability (black) and an indel(s) nearby (grey) are shown for the 36-base reads simulated from the two *D. melanogaster* genotypes (left) and from the *D. melanogaster* and *D. simulans* exomes (right). Each pair of bars results from aligning reads to either a single reference genome (Ref) or both the maternal and paternal genomes (M + P) with zero (0), one (1), two (2), or three (3) mismatches allowed. The two *D. melanogaster* genotypes compared did not include any indels, as described in the main text.

We focused on measures of relative ASE for individual sites in this study, but most researchers are more interested in relative ASE for individual exons and/or genes. The major consequence of excluding sites based on the density of differentiating sites, mappability, and/or indels is that fewer allele-specific reads will be successfully mapped for each exon and for each gene. After filtering based on the number of neighboring differentiating sites, we found that 46.6%-86.9% and 8.3%-50.5% of differentiating sites were retained in the 36-base intra- and interspecific simulations, respectively, when the reads were aligned to a single reference genome and one, two, or three mismatches were allowed (Figure 6). By comparison, 81.8%-91.8% and 66.3%-95.2% of *exons* contained at least one of these reliable differentiating sites when the same alignment conditions were used in the intra- and interspecific simulations, respectively. Excluding additional differentiating sites with imperfect mappability in both datasets, as well as sites with one or more nearby indels in the intraspecific dataset, had little effect on the proportion of differentiating sites and exons retained (Figure 6). The retention of more differentiating sites and exons in the intraspecific simulation than in the interspecific simulation (Figure 6) is consistent with the lower sequence divergence within than between species. Analyses using real and simulated reads to compare the same sets of alleles retain the same sites and exons when aligned to the same reference genome because differentiating sites are excluded based only on the genome sequence(s).

## Conclusions

RNA-seq is a powerful tool for measuring ASE on a genomic scale; however, a systematic bias occurs when reads from a heterozygous individual are aligned to a single reference genome [14]. We found that this systematic bias is predominantly caused by additional differentiating sites located near the focal differentiating site that interfere with read alignment. A similar bias toward the reference allele is caused by the presence of an indel near the focal differentiating site. Differences between alleles in mappability (i.e. the ability to align a read uniquely within the genome) also contribute to inaccuracy of ASE, but do not systematically favor one allele or the other across the genome.

Using both simulated and real sequencing data, we found that sites affected by the systematic bias toward the reference allele could be identified and excluded prior to estimating ASE based on the density of differentiating sites. The precise density at which neighboring differentiating sites became problematic depended on the number of mismatches allowed during the alignment of sequencing reads. After excluding these biased sites, as well as those affected by imperfect mappability and/or an indel(s) nearby, we found that RNA-seq data aligned to a single reference genome produced measures of relative ASE that were comparable to those resulting from separately aligning the same reads to allele-specific maternal and paternal genomes. Furthermore, we showed that excluding these problematic sites did not preclude measuring relative ASE for most exons, although the most rapidly evolving exons are expected to be preferentially eliminated. By identifying the specific factors causing erroneous measures of relative allele-specific expression reported in prior work and determining the relative impact of these factors on these measures, results from this study are expected to foster further improvements in methods for quantifying relative allele-specific expression.

## Methods

### Generating allele-specific short reads comparing *D. melanogaster* genotypes in silico

Simulating an allele-specific RNA-seq experiment requires variability to differentiate alleles and a set of clearly defined transcriptional units from which to generate allele-specific reads. Using data from the Drosophila Genetic Reference Panel (DGRP), we examined site-specific sequence information from a single highly-inbred line (“line_40”) isolated from an outbreeding population of *Drosophila melanogaster*. This specific line was chosen because it had the fewest sites with evidence of residual heterozygosity. Sequence information from this line was compared to the current build of the *D. melanogaster* genome (*dm3*), and sites that differed from this reference genome were retained as sites differentiating the *dm3* and “line_40” alleles, referred to as the reference and alternative alleles, respectively.

Because RNA-seq experiments collect sequence information from the transcribed genome, we chose to generate reads from constitutive exons in *D. melanogaster*[15]. These constitutive exons are defined as those present in all alternatively-spliced transcripts for a particular gene. We filtered out overlapping regions of exons located on opposite strands to avoid ambiguity. Starting from the 5’ end of each exon, we generated 36- and 50-base reads offset by a single base in the 3’ direction, for the reference and alternative alleles and in each strand orientation, creating a complete set of all possible allele-specific and strand-specific reads. This ensured that reads from each allele were present in equal abundance. Because the reference and alternative alleles differed only at these predefined differentiating sites, only reads overlapping these sites had the possibility to be informative for relative ASE.

### Quantifying allelic abundance in simulated RNA-seq data

All alignments were performed using Bowtie v0.12.7 [10], requiring that reads align uniquely to the genome (bowtie -f -m 1 -v [0,1,2,3] --best). Alignments were processed using SAMtools v0.1.18 [13] (samtools view -S -b -T; samtools sort; samtools mpileup -f), which generates site-specific allele frequencies using overlapping reads (read pileup). ASE was quantified using custom Perl and R scripts (available upon request), and any deviation from equal allelic abundance was considered allelic imbalance.

Initially, we aligned the simulated reads to the *D. melanogaster* (*dm3*) reference genome. Since reads generated from the alternative allele overlapping a differentiating site will have at least a single base mismatch to the reference genome, we successively allowed one (-v 1), two (-v 2), or three (-v 3) mismatches, but still required unique alignment to the reference genome (-m 1). Although the -v parameter assesses mismatches for the length of the entire read, and has an upper limit of three, an alternative parameter -n allows additional mismatches outside of a specified region at the beginning of each read, called a seed. To allow a fourth mismatch for the 50-base reads, we specified a 36-base seed region with up to three mismatches and increased the maximum sum of mismatch quality scores across the entire read to 161, since base quality scores for FASTA reads are assumed to equal 40 (bowtie -f -n 3 -e 161 -l 36 -m 1 --best). After each alignment was performed, we considered only reads overlapping the previously defined differentiating sites. We then quantified relative allelic abundance by determining whether or not each overlapping read at these sites matched the reference or the alternative alleles. These summed counts represented our measures of relative allelic abundance at each differentiating site.

Next, we aligned the same allele-specific reads independently to the aforementioned reference genome and the edited copy of the reference genome representing the alternative allele (bowtie -f -m 1 -v 0 --best). As described above, this alternative genome was obtained by editing the bases at differentiating sites to match the fixed genotypes from the DGRP “line_40” sequencing data. No mismatches were allowed when aligning simulated reads to either allele-specific genome. This allowed us to determine, for any read, whether or not it aligned uniquely to one or the other allele-specific genome. We posited that reads aligning uniquely to one or the other allele-specific genome was evidence that that read was allele-specific, while reads aligning equally well to both genomes was not. To measure relative ASE at each differentiating site, we counted the number of reads overlapping differentiating sites that aligned uniquely to only one of the allele-specific genomes and summed these counts for each allele.

### Measuring number of neighboring differentiating sites and mappability across genomes

After quantifying allelic abundance at each differentiating site, we calculated the maximum number of other sites showing differences between alleles contained within any of the possible k-base reads, where k = simulated read length (either 36- or 50-bases). For each genome, we used the GEM-mappability tool from the GEM library build 475 [26] to measure genome mappability, or the ability for a read from a particular location to uniquely align to a genome. For the simulated and real data, we measured mappability for the appropriate read length (either 36 or 50 bases), allowing zero, one, two, or three mismatches, with default parameters (gem-mappability -l [36,50] -m [0,1,2,3]). Mappability for individual sites was calculated using the reciprocal frequency of the number of locations a read beginning at that site would align to in the genome. To calculate mappability scores for differentiating sites, we averaged mappability for all read positions that overlapped each differentiating site [26].

### Quantifying relative ASE in an F1 hybrid between *D. melanogaster* and *D. simulans*

To assess the accuracy of allele-specific abundance inferred from real sequencing data, we used published 36-base Illumina reads from genomic DNA extracted from a pool of female F1 hybrids between laboratory strains of *D. melanogaster* and *D. simulans* (Berlin: BDSC 8522 and C167.4: BDSC 4736, respectively; [16]). We restricted our analysis to the first mate of this set of paired-end reads, combining reads from all three technical replicates. We used the custom set of 60,040 orthologous exon sequences (exomes) between *D. melanogaster* and *D. simulans* developed in Graze et al. [16] for the reference and alternative genomes*.* We also used these sequences to simulate and analyze 36-base reads comparing *D. melanogaster* and *D. simulans* alleles in the same manner outlined above for the two *D. melanogaster* genotypes.

We first performed a pairwise alignment for each orthologous pair of exons using the Fast Statistical Alignment v1.15.7 software [28] with default parameters (fsa --stockholm). We used custom Perl scripts to identify 1,130,435 sites that could differentiate these two alleles as well as to identify regions of the exome present in one allele but not the other (indels).

We then aligned the Illumina reads to the *D. melanogaster* exome, requiring unique alignment to a single location and allowing one, two, or three mismatches. We also aligned the same reads independently to the *D. melanogaster-* and *D. simulans*-specific exomes, masking indels identified by the pairwise alignments. After each of these alignments, we quantified ASE, measured the density of differentiating sites, and determined the mappability to each genome using the same strategies described above for the simulated data. We performed binomial exact tests for differentiating sites with 20 or more overlapping reads, controlling the false discovery rate at 0.05 to correct for multiple comparisons.

## Abbreviations

ASE: Allele-specific expression; DGRP: Drosophila Genetic Reference Panel; qPCR: Quantitative polymerase chain reaction.

## Competing interests

The authors declare that they have no competing interests.

## Authors' contributions

KS, JC, and PW jointly conceived of the study and collaborated on the study design. KS performed all simulations, ran all analyses, and prepared all figures. KS and PW drafted the manuscript with critical input from JC. All authors read and approved the final manuscript.

## Supplementary Material

Additional file 1 **Constitutive exons from the sequenced strain of *Drosophila melanogaster*(*dm3*).** This set of exons was developed as described in McManus *et al.*[15]. We excluded overlapping regions in exons located on opposite strands of DNA from consideration. Click here for file

Additional file 2 **The density of differentiating sites affects measures of relative ASE when simulated reads are mapped to the alternative genome.** Relative ASE was measured by aligning simulated reads to an alternative genome (“line_40”) allowing one mismatch. The number of neighboring differentiating sites is shown on the x-axis, describing the maximum number of other sites that differ between the two alleles in any potential 36-base read overlapping the focal differentiating site. The y-axis shows the proportion of reads that were assigned to the reference allele for each differentiating site, summarized in box plots where the width of each box is proportional to the number of sites in that class. A proportion of 0.5 (indicated with a red dotted line in each panel) is expected if all reads overlapping a differentiating site are correctly assigned to alleles. The pie chart inset reflects the total number of differentiating sites that showed equal (white) and unequal (grey) abundance of reads assigned to each allele. Click here for file

Additional file 3 **36- and 50-base sequence reads produced comparable measures of relative ASE when a similar ratios of mismatches to bases in a sequence read is allowed.** Relative ASE was measured for 36- and 50-base reads simulated from the two *D. melanogaster* genomes by aligning simulated reads to the single reference *D. melanogaster* genome. Three mismatches were allowed for 36-base reads (A), which is 0.083 mismatches per base, and four mismatches were allowed for 50-base reads (B), which is 0.080 mismatches per base. The number of neighboring differentiating sites is shown on the x-axis, describing the maximum number of other sites that differ between the two alleles in any potential 36-base (A) or 50-base (B) read overlapping the focal differentiating site. The y-axis shows the proportion of reads that were assigned to the reference allele for each differentiating site, summarized in box plots where the width of each box is proportional to the number of sites in that class. A proportion of 0.5 (indicated with a red dotted line in each panel) is expected if all reads overlapping a differentiating site are correctly assigned to alleles. The pie chart inset reflects the total number of differentiating sites that showed equal (white) and unequal (grey) abundance of reads assigned to each allele. Click here for file
