# Supplementary figures and images for: Sources of bias in measures of allele-specific expression derived from RNA-seq data aligned to a single reference genome
Source: BMC Genomics. 2013 Aug 7;14:536. doi: 10.1186/1471-2164-14-536 (PMC3751238; doi:10.1186/1471-2164-14-536)

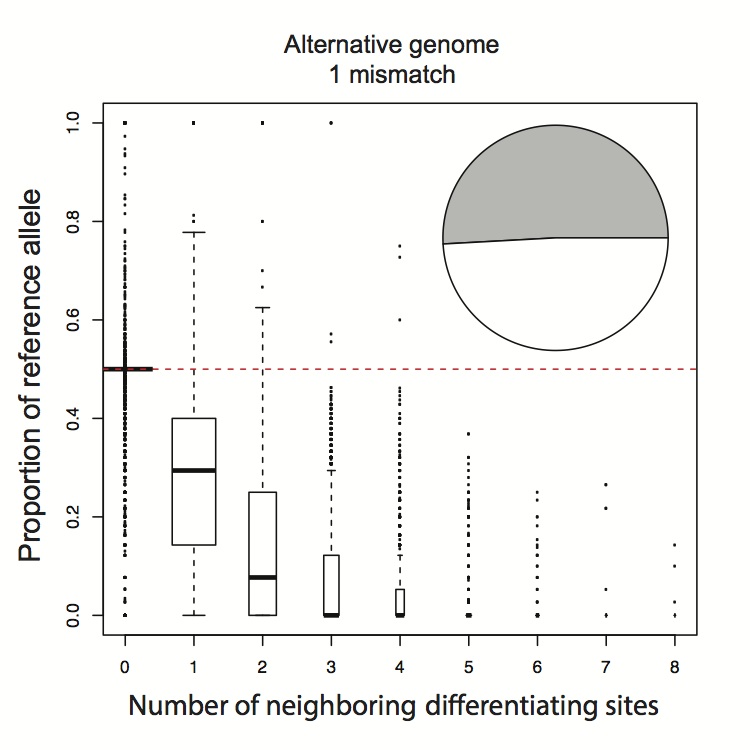

Supplement: Additional file 2 — The density of differentiating sites affects measures of relative ASE when simulated reads are mapped to the alternative genome. Relative ASE was measured by aligning simulated reads to an alternative genome (“line_40”) allowing one mismatch. The number of neighboring differentiating sites is shown on the x-axis, describing the maximum number of other sites that differ between the two alleles in any potential 36-base read overlapping the focal differentiating site. The y-axis shows the proportion of reads that were assigned to the reference allele for each differentiating site, summarized in box plots where the width of each box is proportional to the number of sites in that class. A proportion of 0.5 (indicated with a red dotted line in each panel) is expected if all reads overlapping a differentiating site are correctly assigned to alleles. The pie chart inset reflects the total number of differentiating sites that showed equal (white) and unequal (grey) abundance of reads assigned to each allele. [file 1471-2164-14-536-S2.jpeg]

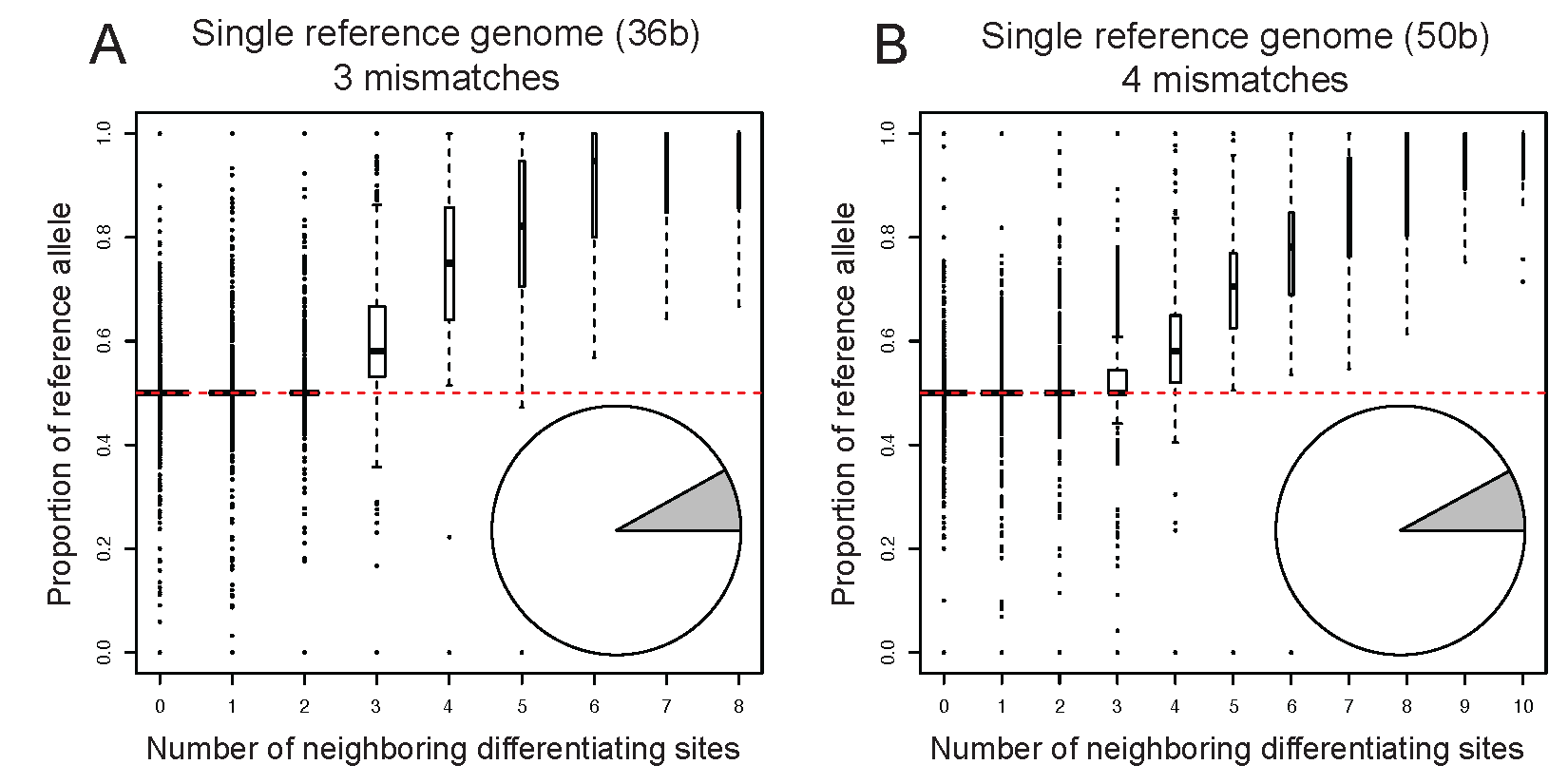

Supplement: Additional file 3 — 36- and 50-base sequence reads produced comparable measures of relative ASE when a similar ratios of mismatches to bases in a sequence read is allowed. Relative ASE was measured for 36- and 50-base reads simulated from the two D. melanogaster genomes by aligning simulated reads to the single reference D. melanogaster genome. Three mismatches were allowed for 36-base reads (A), which is 0.083 mismatches per base, and four mismatches were allowed for 50-base reads (B), which is 0.080 mismatches per base. The number of neighboring differentiating sites is shown on the x-axis, describing the maximum number of other sites that differ between the two alleles in any potential 36-base (A) or 50-base (B) read overlapping the focal differentiating site. The y-axis shows the proportion of reads that were assigned to the reference allele for each differentiating site, summarized in box plots where the width of each box is proportional to the number of sites in that class. A proportion of 0.5 (indicated with a red dotted line in each panel) is expected if all reads overlapping a differentiating site are correctly assigned to alleles. The pie chart inset reflects the total number of differentiating sites that showed equal (white) and unequal (grey) abundance of reads assigned to each allele. [file 1471-2164-14-536-S3.png]
